# Is step width decoupled from pelvic motion in human evolution?

**DOI:** 10.1038/s41598-020-64799-3

**Published:** 2020-05-08

**Authors:** Michelle Kikel, Rachel Gecelter, Nathan E. Thompson

**Affiliations:** 1 New York Institute of Technology, College of Osteopathic Medicine, Old Westbury, NY 11568 USA; 2 Department of Anatomy, NYIT College of Osteopathic Medicine, Old Westbury, NY 11568 USA

**Keywords:** Biological anthropology, Musculoskeletal system

## Abstract

Humans are the only primate that walk bipedally with adducted hips, valgus knees, and swing-side pelvic drop. These characteristic frontal-plane aspects of bipedalism likely play a role in balance and energy minimization during walking. Understanding when and why these aspects of bipedalism evolved also requires an understanding of how each of these features are interrelated during walking. Here we investigated the relationship between step width, hip adduction, and pelvic list during bipedalism by altering step widths and pelvic motions in humans in ways that both mimic chimpanzee gait as well as an exaggerated human gait. Our results show that altering either step width or pelvic list to mimic those of chimpanzees affects hip adduction, but neither of these gait parameters dramatically affects the other in ways that lead to a chimpanzee-like gait. These results suggest that the evolution of valgus knees and narrow steps in humans may be decoupled from the evolution of the human-like pattern of pelvic list. While the origin of narrow steps in hominins may be linked to minimizing energetic cost of locomotion, the origin of the human-like pattern of pelvic list remains unresolved.

## Introduction

**Figure 1:**
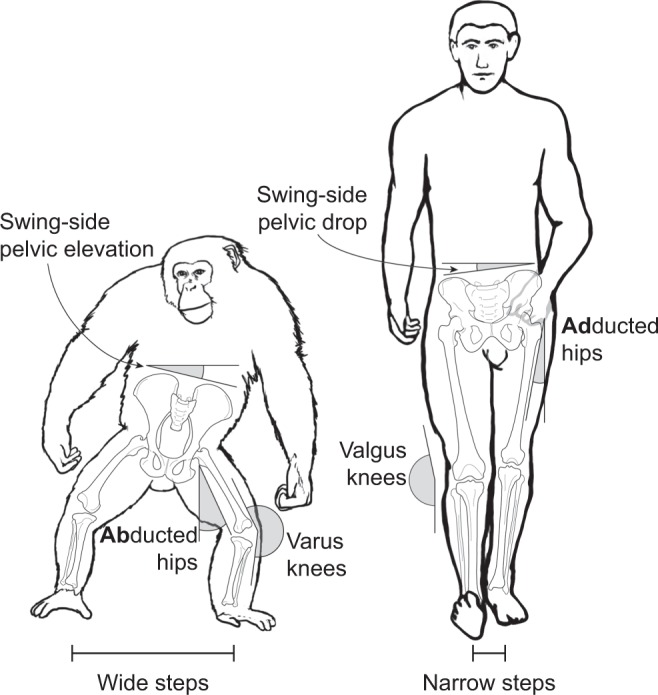
Major frontal-plane differences in hind/lower limb kinematics between chimpanzees and humans. Note that some kinematics have been slightly exaggerated for clarity.

As the only habitually bipedal primates, humans display a suite of unique gait attributes not seen during facultative bipedalism in other primates. Several of these traits relate to the need to maintain balance and stability in the frontal plane (Fig. 1). Notably, at the pelvis, humans display a unique pattern of swing-side pelvic drop and concomitant hip adduction during stance phase. Conversely, facultative bipedalism in chimpanzees and macaques involves swing-side pelvic elevation as well as highly abducted hip positions^1–3^. By virtue of adducted hips and valgus knees (via the presence of an osteological femoral bicondylar angle) humans also display step widths that are approximately 2.5–3.3 times narrower than those measured for non-human apes^4^.

When these characteristic aspects of human-like bipedalism emerged in the hominin fossil record has long been a matter of debate. Valgus knees and femoral bicondylar angles, indicative of narrow steps and some level of hip adduction during bipedalism, are the clearest morphological trait indicative of these locomotor characteristics. Human-like bicondylar angles are present as early as ~3.6 million years ago (mya) from *Australopithecus afarensis* (KSD-VP-1/1)^5^. Direct morphological evidence of human-like swing-side pelvic drop however, is less clear. Many have argued based on aspects of iliac blade and/or femoral head and neck morphology that human-like pelvic and hip kinematics may have been present as early as *Au. afarensis*^6–9^, *Ardipithecus ramidus*^10,11^, or perhaps even earlier^12,13^. These arguments, however, have been vigorously debated^4,14–19^.

Understanding when and why these locomotor characteristics evolved also requires knowledge about their function during bipedalism. Bicondylar angles, and the narrow steps resulting therefrom, have been shown to reduce mediolateral oscillations of the center of mass (CoM)^4,20^, and thereby reduce metabolic energy expenditure during walking^20^. The selective advantage of swing-side pelvic drop is unresolved. Classically, it has been suggested that pelvic drop serves to reduce vertical oscillations of the CoM^21^. More recent data refutes this idea as pelvic list has a minimal effect on vertical CoM oscillations^22–24^. Alternatively, swing-side pelvic drop may be a consequence of changes to other biomechanical variables during bipedalism. Pelvic list, hip abduction, and step width are all part of body coordination in the frontal plane^25,26^. Thus the selective pressure to change one feature, for instance step width, could be expected to cause correlated change in hip abduction and pelvic list or vice versa. In order words, the origin of narrow steps in hominins may have driven changes toward the human-like pattern of pelvic list.

Here we investigated the hypothesis that step width is linked to the unique pattern of human hip adduction and pelvic motion during bipedalism. We tested this hypothesis in two ways; first by having humans walk with altered step widths, including chimpanzee-like wide steps^4^. If step width and pelvic motion are linked, we expected that wide steps should cause humans to adopt a pattern of chimpanzee-like swing-side pelvic elevation and hip abduction. Second, we had subjects walk with altered pelvic kinematics, including bipedal-primate-like swing-side pelvic elevation^3^. We expected that, if pelvic motion and step width are linked, pelvic elevation should increase step widths to a value similar to those seen in bipedal primates.

## Methods

### Experimental subjects and protocol

All experimental protocols were approved by the New York Institute of Technology’s Internal Review Board and were performed in accordance with all relevant guidelines and regulations and with the principles outlined in the Declaration of Helsinki. Subjects provided informed written consent prior to experiments. Three-dimensional kinematic data were recorded on ten human subjects (five females, five males; body mass = 68.5 ± 11.5 kgs; age = 25.4 ± 1.9 years; complete subject data are reported in Supplementary Table S1) during walking. All subjects walked on an AMTI (Watertown, MA, U.S.A.) treadmill at 1.0 m/s. Subjects were instructed to walk with varying step width conditions (normal, narrow, wide) and varying pelvic conditions (normal, exaggerated pelvic drop, pelvic elevation). For the narrow step width condition, subjects were instructed to walk with their feet falling along a straight line. To achieve wide step widths, subjects walked placing their feet outside of two strings placed on the treadmill (similar to^20^). The distance between the strings was calculated based on subject’s normal step width and set to achieve a step width of approximately three times the normal width. This represents the approximate difference in relative step widths between humans and chimpanzees^4^. Subjects were given ample time to practice walking with the various gait conditions prior to data recording. Ten strides were analyzed for each condition for each subject.

### Kinematic data collection and methodology

**Figure 2:**
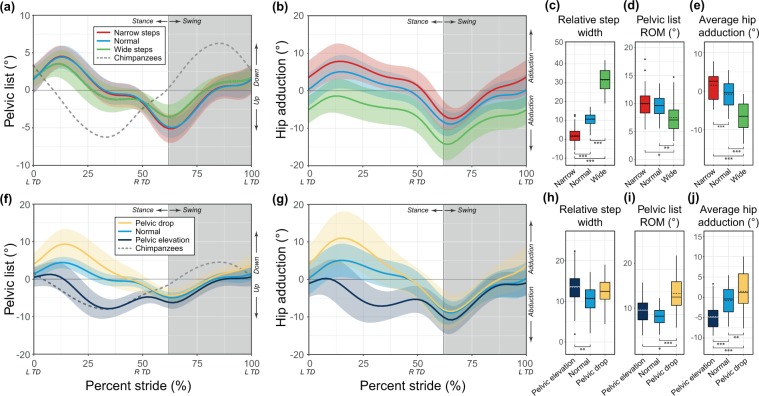
Pelvic list (**a,f**) and hip adduction (**b,g**) for varying step widths (top row) and pelvic conditions (bottom row); all strides start and end at left touchdown (L TD). Note the difference in y-axis scale of (**a**). Box-and-whisker plots show relative step width (**c,h**), pelvic list range of motion (ROM, **d,i**), and average hip adduction (**e,j**). Boxes represent the first through third quartiles and the solid and dashed lines within the boxes represent the median and mean respectively. Whiskers represent 1.5 times the interquartile range, and dots represent outliers. Chimpanzee pelvic list (dashed grey line of **a,f**) are drawn from^4^. Light blue is normal walking, red and green represent narrow and wide step widths, respectively, and gold and dark blue represent pelvic drop and pelvic elevation, respectively. Asterisks represent post-hoc pairwise significance between conditions from the linear mixed models as in Table 1.

Kinematic data were collected using a 12-camera Vicon motion capture system (Vicon Motion Systems Ltd., Oxford, U.K.) at 150 frames-per-second and using the full body Plug-In Gait marker set (based on the Newington/Helen Hayes gait model)^27,28^. Marker positions were filtered using a Woltring filter^29^. Pelvic and hip angles were calculated using the default Plug-In Gait analysis and were taken directly from Vicon Nexus (v2.9, Vicon Motion Systems) and compiled in Polygon and ProCalc (Vicon Motion Systems) for further analysis. For plots of pelvic list over the stride, the mean pelvic list for two chimpanzee subjects, previously published and recorded using similar methods^4^, was also included for reference (Fig. 2a,f; dashed line).

Step widths were calculated by establishing a line between the positions of the left heel marker at two consecutive ipsilateral heel strikes, and subtracting the mediolateral distance of the right heel marker at contralateral heel strike from this line. Step width was reported both absolutely (in cm) and relatively, by dividing step width by standing lower limb length (from greater trochanter to the ground).

### Statistics

All statistical comparisons were performed using linear mixed models (LMMs) in the package lme4^30^ for R 3.6.1^31^. Two classes of LMMs were conducted based on the fixed effect of either step width condition or pelvic condition. Subject was included as a random factor with subject-specific slopes and intercepts. Each kinematic variable (Table 1) was set as the response variable, and each LMM was tested against a null model (excluding the fixed effect) to test for model significance. All full-to-null model comparisons were significant. Finally, for each kinematic variable, post-hoc pairwise comparisons within conditions (either step width or pelvic condition) were conducted using the package emmeans^32^ and Tukey family-wise error rate corrections.

**Table 1:** Kinematic variables for all conditions and significance of pair-wise comparisons from the linear mixed models.

Step Condition	Normal		Narrow steps		Wide steps		Pelvic elevation		Pelvic drop		Step condition sig			Pelvic condition sig		
	mean	*st. dev*.	mean	*st. dev*.	mean	*st. dev*.	mean	*st. dev*.	mean	*st. dev*.	N-NS	N-WS	NS-WS	N-PE	N-PD	PE-PD
Age (years)	25.4	*1.9*	—	—	—	—	—	—	—	—						
Weight (kg)	68.5	*11.5*	—	—	—	—	—	—	—	—						
Lower limb length (cm)	87.0	*5.8*	—	—	—	—	—	—	—	—						
Step width (cm)	9.2	*2.9*	1.9	*3.4*	27.2	*5.6*	11.7	*3.1*	10.7	*2.7*	***	***	***	**	—	—
Relative step width	10.6	*3.2*	2.1	*3.9*	31.4	*6.3*	13.4	*3.5*	12.4	*3.3*	***	***	***	**	—	—
**Pelvic list**																
Average (°)	−0.1	*1.2*	−0.1	*1.3*	0.0	*1.1*	−3.3	*1.7*	1.9	*2.2*	—	—	—	***	**	***
Range of motion (°)	9.6	*1.8*	9.9	*2.2*	7.5	*2.6*	11.0	*2.8*	14.7	*3.9*	—	*	**	—	***	*
Maximum (°)	4.5	*1.5*	4.7	*1.4*	3.7	*1.7*	2.3	*2.7*	10.0	*3.8*	—	*	—	*	***	***
Minimum(°)	−5.1	*1.4*	−5.2	*1.9*	−3.7	*1.6*	−8.7	*2.0*	−4.6	*2.1*	—	*	*	***	—	***
**Hip adduction**																
Average (°)	−0.8	*3.3*	1.5	*3.3*	−6.5	*3.8*	−4.7	*3.1*	1.5	*4.5*	***	***	***	***	**	***
Range of motion (°)	14.5	*3.9*	16.2	*3.9*	13.5	*4.1*	13.6	*5.5*	21.0	*5.5*	*	—	*	—	**	**
Maximum (°)	5.3	*4.4*	8.2	*4.6*	−1.1	*4.3*	1.9	*4.8*	12.0	*6.7*	***	***	***	*	***	***
Minimum (°)	−9.2	*3.2*	−8.0	*3.3*	−14.5	*4.3*	−11.7	*3.8*	−9.0	*4.8*	*	***	***	***	—	**

Abbreviations: sig, significance; st dev, standard deviation; N, normal walking; NS, narrow steps; WS, wide steps; PE, pelvic elevation; PD, pelvic drop. Asterisks indicate significance at the 0.05 (*), 0.01 (**), or 0.001 (***) level for post-hoc pairwise comparisons resulting from the linear mixed models.

## Results

Walking with wide steps increased subjects’ relative steps widths to 3.0 times that of normal walking (31.4 ± 6.3 versus 10.6 ± 3.2, *p* < 0.001; Fig. 2c; Table 1), similar to the difference in relative step width between humans and bipedal chimpanzees (3.3 times that of humans^4^) as intended. Narrow steps decreased step widths from 9.2 ± 2.9 to 1.9 ± 3.4 centimeters (*p* < 0.001; Table 1). Predictably, narrow and wide steps led to increased and decreased average hip adduction, respectively (narrow: 1.5 ± 3.3°, normal: −0.8 ± 3.3°, wide: −6.5 ± 3.8°, *p* < 0.001 for all pairwise comparisons; Fig. 2b,e; Table 1). However, the pattern of hip adduction was largely the same between conditions, though narrow steps displayed a slightly larger range of motion (ROM) than either normal or wide step widths (narrow: 16.2 ± 3.9°, normal: 14.5 ± 3.9°, wide: 13.5 ± 4.1°, *p* < 0.05 between narrow steps and other step width conditions; Table 1). The pattern of pelvic list was largely unaffected by step width (Fig. 2a). Pelvic list ROM at narrow and normal step widths were nearly identical (narrow: 9.9 ± 2.2°, normal: 9.6 ± 1.8°, *n.s*.; Table 1). Only wide steps differed in having a pelvic list ROM that was reduced by approximately 2° from the normal walking condition (normal: 9.6 ± 1.8°, wide steps: 7.5 ± 2.6°, *p* < 0.05; Fig. 2d; Table 1); however the pattern of motion remained distinctly human-like (compared with dashed line in Fig. 2a representing chimpanzee pelvic list).

For pelvic conditions, both target conditions achieved the intended results during stance phase, while swing phase kinematics were largely unaltered (Fig. 2f). The pelvic elevation condition resulted in stance-phase pelvic list that approached chimpanzee-like pelvic list (compare dark blue and dashed lines in Fig. 2f). The pelvic drop condition resulted in increased downward pelvic list during stance phase (Fig. 2f) and an increased ROM (pelvic drop: 14.7 ± 3.9°, normal: 9.6 ± 1.8°, *p* < 0.001; Fig. 2i; Table 1). Average hip adduction angles were also significantly increased (pelvic drop) and decreased (pelvic elevation) compared with normal walking (pelvic drop: 1.5 ± 4.5°, normal: -0.8 ± 3.3°, pelvic elevation: −4.7 ± 3.1°, *p* < 0.01 between pelvic conditions; Fig. 2g,j; Table 1). Both experimental conditions displayed increased relative step widths compared with normal walking (pelvic drop: 12.4 ± 3.3, normal: 10.6 ± 3.2, pelvic elevation: 13.4 ± 3.5; Fig. 2h; Table 1), though only significantly so in the pelvic elevation condition (*p* < 0.01 normal and pelvic elevation).

## Discussion

Walking bipedally with chimpanzee-like wide steps did not cause human pelvic kinematics to drastically vary from the unique human pattern. It did reduce the normal range of motion by 22%, or about 2°, however the pattern of motion remained distinctly human-like (Fig. 2a). Altering step widths did lead to significant and predictable changes in average hip adduction, but our data suggest that the human-like pattern of pelvic list itself is largely robust to changes in step width. Similarly, walking with chimpanzee-like pelvic list (i.e., pelvic elevation) did slightly increase step widths, but not drastically so (~0.3 times larger as opposed to the 3.3 times larger steps of chimpanzees). Furthermore, both the pelvic drop and pelvic elevation conditions led to increased step widths, suggesting that wider steps may result from prescribing a non-normal pattern of lower limb kinematics. Stance-phase hip motion however was significantly altered between pelvic conditions, with pelvic elevation leading to more abduction and exaggerated pelvic drop to more adduction.

Taken together, our results show that experimentally altering either pelvic motions or step widths does have a strong effect on hip adduction. Yet, neither of these effects are so extreme that they cause major deviations in underlying step widths (in the case of altered pelvic conditions) nor the pattern of pelvic list (in the case of altered steps). Therefore, the evolution of femoral bicondylar angles and valgus knees in hominins and the associated narrow step widths may not have evolved in concert with the appearance of human-like swing-side pelvic drop. These two characteristic features of human bipedalism may have evolved at different times in hominin evolution, and for different reasons. It is clear that valgus knees and thus narrow steps emerged fairly early in hominin evolution, at least by ~3.6 mya in *Au. afarensis*^5^ though perhaps earlier. Narrow, human-like, step widths engender a clear energetic advantage compared with wide steps^20,33^ and femoral bicondylar angles in early hominins represent the presence of this energetic benefit.

However, the biomechanical reason underlying the change from a primate-like pattern of pelvic list to a human-like pattern is still unresolved. Swing-side pelvic elevation during facultative bipedalism in primates may function to aid in foot clearance^2^, increase stride length^2^, or shift the center of mass towards the stance side foot^2,4^. Furthermore, during quadrupedalism, mammals utilize swing-side pelvic elevation (i.e., rotation of the swing-side acetabulum cranially^34,35^). It is possible that the retention of similar pelvic kinematics during bipedalism in non-human primates points to some degree of neuromuscular conservatism retained from quadrupedal gait patterns (e.g.^36^).

The current experimental evidence suggests that it is only within hominins that a bipedal gait entailing swing-side pelvic drop evolved^3^. Classical descriptions of human bipedalism attribute stance-phase pelvic drop to minimizing center of mass vertical displacements, which could reduce energy requirements^21^. More recent studies have shown that pelvic list only has a small^22,24^, and perhaps negligible^23^ effect on vertical CoM displacements. Moreover, decreasing vertical CoM displacement likely increases, rather than decreases, the metabolic cost of locomotion^37,38^. Ultimately, the best way to understand when and why a human-like pattern of pelvic list evolved may be through forward dynamic modeling of fossil hominins. The only current forward dynamic model of fossil hominin bipedalism (of the ‘Lucy’ skeleton; *Au. afarensis*) suggests some slight degree of stance-phase hip abduction, but whether or not this was due to stance-phase pelvic elevation was not reported^39^.

Future forward dynamic modeling may also offer a resolution to the biggest limitation of this study, which is that we utilized human subjects walking in ways that targeted non-human primate-like gait parameters. We were fundamentally unable to experimentally alter underlying bicondylar angle and hip morphology within our subjects, and therefore could not directly investigate the effect of morphology. Thus, the earliest hominins may not have displayed the same relationship between variables that we measured here in modern humans. Forward dynamic simulations may allow for further investigation of step width, pelvic motion, and hip adduction, while simultaneously modeling non-human skeletal morphology.

The data herein suggest that human-like pelvic list and valgus knees (and corresponding narrow steps) represent two different biomechanical phenomenon which may not be directly coupled in human evolution. While this would not preclude the simultaneous appearance of both factors, it does open the possibility that narrow steps and valgus knees may have temporally preceded the human-like pelvic kinematics, or vice versa. It is well-established that relatively narrow, human-like steps offer an energetic advantage for bipeds^20,33^. The specific reasons why humans, and likely some hominins, utilize swing-side pelvic drop remains an open question.

## Supplementary information

Supplementary Information.

Supplementary Information2.

## Data Availability

The dataset for this work is available as Supplementary Table 1.

## References

[1] Jenkins FA (1972). Chimpanzee bipedalism: cineradiographic analysis and implications for the evolution of gait. Science.

[2] O’Neill MC (2015). Three-dimensional kinematics of the pelvis and hind limbs in chimpanzee (*Pan troglodytes*) and human bipedal walking. J. Hum. Evol..

[3] O’Neill MC, Demes B, Thompson NE, Umberger BR (2018). Three-dimensional kinematics and the origin of the hominin walking stride. J. R. Soc. Interface.

[4] Thompson NE, Demes B, Holowka NB, O’Neill MC (2018). Step width and frontal plane trunk motion in bipedal chimpanzee and human walking. J. Hum. Evol..

[5] Haile-Selassie Y (2010). An early *Australopithecus afarensis* postcranium from Woranso-Mille, Ethiopia. Proc. Natl. Acad. Sci. USA.

[6] Lovejoy CO, Heiple KG, Burstein AH (1973). The gait of *Australopithecus*. Am. J. Phys. Anthropol..

[7] Lovejoy CO (1988). Evolution of human walking. Sci. Am..

[8] Lovejoy CO, Meindl RS, Ohman JC, Heiple KG, White TD (2002). The Maka femur and its bearing on the antiquity of human walking: applying contemporary concepts of morphogenesis to the human fossil record. *Am*. J. Phys. Anthropol..

[9] Lovejoy, C. O., Latimer, B. M., Spurlock, L. & Haile-Selassie, Y. The pelvic girdle and limb bones of KSD-VP-1/1. In *The Postcranial Anatomy of Australopithecus afarensis* (eds. Haile-Selassie, Y. & Su, D. F.) 155–178 (Springer, 2016).

[10] Lovejoy CO, Suwa G, Simpson SW, Matternes JH, White TD (2009). The great divides: *Ardipithecus ramidus* reveals the postcrania of our last common ancestors with African apes. Science.

[11] Lovejoy CO, Suwa G, Spurlock L, Asfaw B, White TD (2009). The pelvis and femur of *Ardipithecus* ramidus: the emergence of upright walking. Science.

[12] Böhme M (2019). A new Miocene ape and locomotion in the ancestor of great apes and humans. Nature.

[13] Richmond BG, Jungers WL (2008). *Orrorin tugenensis* femoral morphology and the evolution of hominin bipedalism. Science.

[14] Berge, C. Quelle est la signification fonctionnelle du pelvis très large de *Australopithecus afarensis* (AL 288-1)? In *Origine(s) de la Bipédie chez les**Hominidés* (eds. Coppens, Y. & Senut, B.) 113–119 (CNRS, 1991).

[15] Ruff, C. B. Evolution of the hominid hip. in *Primate Locomotion: Recent Advances* (eds. Strasser, E., Fleagle, J. G., Rosenberger, A. L. & McHenry, H. M.) 449–469 (Plenum Press, 1998).

[16] Ruff CB, Higgins R (2013). Femoral neck structure and function in early hominins. Am. J. Phys. Anthropol..

[17] Susman, R. L. & Stern, J. T. Locomotor behavior of early hominids: epistemology and fossil evidence. in *Origine(s) de la Bipédie chez les**Hominidés* (eds. Coppens, Y. & Senut, B.) 121–132 (CNRS, 1991).

[18] Stern, J. T. & Susman, R. L. ‘Total morphological pattern’ versus the ‘magic trait’: conflicting approaches to the study of early hominid bipedalism. in *Origine(s) de la Bipédie chez les* Hominidés (eds. Coppens, Y. & Senut, B.) 99–112 (CNRS, 1991).

[19] Stern JT, Susman RL (1983). The locomotor anatomy of *Australopithecus afarensis*. Am. J. Phys. Anthropol..

[20] Donelan JM, Kram R, Arthur D (2001). K. Mechanical and metabolic determinants of the preferred step width in human walking. Proc. R. Soc. B Biol. Sci..

[21] Saunders JB, Inman VT, Eberhart HD (1953). The major determinants in normal and pathological gait. J. Bone Joint. Surg. Am..

[22] Gard SA, Childress DS (1997). The effect of pelvic list on the vertical displacement of the trunk during normal walking. Gait Posture.

[23] Gard SA, Childress DS (2001). What Determines the vertical displacement of the body during normal walking?. J. Prosthet. Orthot..

[24] Della Croce U, Riley PO, Lelas JL, Kerrigan DC (2001). A refined view of the determinants of gait. Gait Posture.

[25] MacKinnon C, Winter DA (1993). Control of whole body balance in the frontal plane during human walking. J. Biomech..

[26] Pandy MG, Lin YC, Kim HJ (2010). Muscle coordination of mediolateral balance in normal walking. J. Biomech..

[27] Davis RB, Õunpuu S, Tyburski D, Gage JR (1991). A gait analysis data collection and reduction technique. Hum. Mov. Sci.

[28] Kadaba MP, Ramakrishnan HK, Wootten ME (1990). Measurement of lower extremity kinematics during level walking. J. Orthop. Res..

[29] Woltring HJ (1986). A FORTRAN package for generalized, cross-validatory spline smoothing and differentiation. Adv. Eng. Softw..

[30] Bates D, Mächler M, Bolker BM, Walker SC (2015). Fitting linear mixed-effects models using lme4. J. Stat. Softw..

[31] R Core Team. R: a language and evironment for statistical computing. Version 3.6.1. R Foundation for Statistical Computing, Vienna Available at, http://www.Rproject.org/ (2019).

[32] Lenth, R., Singman, H., Love, J., Buerkner, P. & Herve, M. *emmeans: estimated marginal means, aka least-squares means*. R package version 1.4.3.01 Available at, https://cran.r-project.org/package=emmeans (2019).

[33] Shorter KA, Wu A, Kuo AD (2017). The high cost of swing leg circumduction during human walking. Gait Posture.

[34] Jenkins FA, Camazine SM (1977). Hip structure and locomotion in ambulatory and cursorial carnivores. J. Zool.

[35] Schmidt M (2005). Quadrupedal locomotion in squirrel monkeys (Cebidae: *Saimiri sciureus*): a cineradiographic study of limb kinematics and related substrate reaction forces. Am. J. Phys. Anthropol..

[36] Shapiro LJ, Jungers WL (1994). Electromyography of back muscles during quadrupedal and bipedal walking in primates. Am. J. Phys. Anthropol..

[37] Ortega JD (2005). Minimizing center of mass vertical movement increases metabolic cost in walking. J. Appl. Physiol..

[38] Gordon KE, Ferris DP, Kuo AD (2009). Metabolic and mechanical energy costs of reducing vertical center of mass movement during gait. Arch. Phys. Med. Rehabil..

[39] Nagano A, Umberger BR, Marzke MW, Gerritsen KGM (2005). Neuromusculoskeletal computer modeling and simulation of upright, straight-legged, bipedal locomotion of *Australopithecus afarensis* (A.L. 288-1). Am. J. Phys. Anthropol..

