# Supplementary material for: Is step width decoupled from pelvic motion in human evolution?
Source: Sci Rep. 2020 May 8;10:7806. doi: 10.1038/s41598-020-64799-3 (PMC7210942; doi:10.1038/s41598-020-64799-3)
Supplement: Supplementary file 1 — Supplementary Information. [file 41598_2020_64799_MOESM1_ESM.pdf]

Supplemental Material for:

Is step width decoupled from pelvic motion in human evolution?

Michelle Kikel[a], Rachel Gecelter[a], Nathan E Thompson[b, *]

[a] New York Institute of Technology, College of Osteopathic Medicine, Old Westbury, NY 11568

[b] Department of Anatomy, NYIT College of Osteopathic Medicine, Old Westbury, NY 11568

* Corresponding Author

Contents:

Supplemental Table S1: Kinematic data for all strides and subjects.
